# The Effects of Breed and Residual Feed Intake Divergence on the Abundance and Active Population of Rumen Microbiota in Beef Cattle

**DOI:** 10.3390/ani12151966

**Published:** 2022-08-03

**Authors:** Yawei Zhang, Fuyong Li, Yanhong Chen, Le-Luo Guan

**Affiliations:** 1 College of Animal Science, Shanxi Agricultural University, Taiyuan 030031, China; ywzhang@sxau.edu.cn; 2 Department of Agricultural, Food and Nutritional Science, University of Alberta, Edmonton, AB T6G 2P5, Canada; lifuyong04105104@gmail.com (F.L.); yanhong@ualberta.ca (Y.C.)

**Keywords:** beef cattle breed, eukaryotes, feed efficiency, rumen microbiota, residual feed intake

## Abstract

**Simple Summary:**

This study assessed the effects of breed and feed efficiency on rumen microbiota in a total of 96 beef steers, with three breeds and divergent residual feed intakes. The abundance and activity of the total bacteria, archaea, protozoa, and fungi in the rumens were estimated by measuring the copy numbers of their respective marker genes at both DNA (abundance) and RNA (activity) levels. Our results evidence the effect of breed on four microbial groups. Past studies have been mainly focused on bacteria and archaea, yet ours is the first study to reveal the effect of breed (host genetics) on rumen eukaryotes, suggesting that host genetics can regulate the rumen microbiota as a whole, highlighting the potential of manipulating and obtaining desirable and efficient rumen microbiota using breeding and genetic selection.

**Abstract:**

To assess the effects of residual feed intake (RFI) and breed on rumen microbiota, the abundance (DNA) and active population (RNA) of the total bacteria, archaea, protozoa, and fungi in the rumen of 96 beef steers from three different breeds (Angus (AN), Charolais (CH), and Kinsella Composite (KC)), and divergent RFIs (High vs Low), were estimated by measuring their respective maker gene copies using qRT-PCR. All experimental animals were kept under the same feedlot condition and fed with the same high-energy finishing diet. Rumen content samples were collected at slaughter and used for the extraction of genetic material (DNA and RNA) and further analysis. There was a significant difference (*p* < 0.01) between the marker gene copies detected for abundance and active populations for all four microbial groups. AN steers had a higher abundance of bacteria (*p* < 0.05) and a lower abundance of eukaryotes (protozoa and fungi, *p* < 0.05) compared to KC steers, while the abundance of protozoa (*p* < 0.05) in the AN cattle and fungi (*p* < 0.05) in the KC cattle were lower and higher, respectively, than those in the CH steers. Meanwhile, the active populations of bacteria, archaea, and protozoa in the KC steers were significantly lower than those in the AN and CH animals (*p* < 0.01). This work demonstrates that cattle breed can affect rumen microbiota at both the abundance and activity level. The revealed highly active protozoal populations indicate their important role in rumen microbial fermentation under a feedlot diet, which warrants further study.

## 1. Introduction

Improvement of feed efficiency for beef cattle has the potential to increase the producer’s profitability and lower the environmental footprint of beef production [1]. Feed efficiency can be affected by many factors, including genetics [2,3], the environment [4], nutrition [5,6], etc. Recent studies have reported that the compositional variations of rumen microbiota, specifically bacteria [7,8,9,10] and archaea [11,12,13], are associated with the feed efficiency of beef cattle. However, previous studies on feed efficiency that were associated with rumen microbes only focused on bacteria and archaea at the DNA level, with a few studies reporting this relationship between active rumen bacteria and archaea with cattle feed efficiency [14,15]. Recent studies have revealed that the different outcomes in bacterial composition, and the relative abundance in the rumen, between DNA and RNA levels when the same samples were assessed based on amplicon sequencing [16], suggested that DNA-based microbial analysis could be biased (false positive) due to the possibility of including DNA from dead cells and the other sources. However, to the best of our knowledge, few studies have directly compared the quantitative differences in rumen bacterial and archaeal communities, quantified by DNA- and RNA-based methods for the same samples.

It is known that rumen microbiota consists of diverse microorganisms, including bacteria, archaea, protozoa, and anaerobic fungi, who work cooperatively to facilitate the digestion of the feed and produce volatile fatty acids (VFA) to supply the host ruminant with up to 70% of their energy and nutrient requirements [17]. Meanwhile, there are significant interactions among different microbial groups at both the compositional and functional levels [18]. However, there are very few studies that have investigated the role of rumen eukaryotes in feed efficiency at both the DNA (abundance) and RNA (active population) levels.

Although diet has been considered the main factor to drive the rumen microbiota shift, more and more evidence has highlighted the effect of the host’s genetics and the composition and relationship with host phenotypes [19,20,21]. A more recent study by Li et al. [2] revealed the potential heritability of some rumen bacterial and archaeal taxa, providing further evidence that host genetics, together with dietary and environmental factors, drives the composition of rumen microbiota. These breed-associated differences in the rumen microbiome represent an opportunity to manipulate specific rumen microbiota, thereby improving feed efficiency through the selective breeding of the hosts. As described above, however, the host’s genetic effects on rumen eukaryotes have not been studied due to their relatively lower population compared to rumen bacteria and archaea [22].

We speculated that cattle breed can affect rumen prokaryotes (bacteria and archaea), and eukaryotes (protozoa and fungi) at both the abundance (DNA) and active population (RNA) levels. Therefore, in the current study, the marker gene copies of four ruminal microbial groups in the rumen of 96 beef steers belonging to three breeds, who were fed the same feedlot diet, were quantified at both the DNA and RNA level using quantitative real-time PCR. Meanwhile, an important objective of this study was to gain a better understanding of the differences between the techniques that use different genetic materials (DNA or RNA) and how they affect the interpretation of microbiota-quantitative data. In addition, the relationships between the four groups of rumen microbiota were investigated in this study. Moreover, these cattle were selected from a large population (460 cattle) based on their residual feed intake (RFI) ranking. RFI is one of the measures for feed efficiency [1], which is defined as the difference between an animal’s actual and predicted feed intake, thus animals with a low RFI are efficient, while animals with a high RFI are inefficient [23]. Therefore, we further investigated the effect of RFI and/or interactions between RFI and breed on rumen microbiota, especially eukaryotes.

## 2. Materials and Methods

### 2.1. Animal Experiments and Sample Collection

During two consecutive years, from 2014 to 2015, a total of 460 steers belonging to three breeds, including Angus (AN), Charolais (CH), and a crossbred Kinsella Composite (KC), were raised under the same feedlot conditions at the Roy Berg Kinsella Research Ranch, University of Alberta. The KC population was bred from multiple breeds, including Angus, Charolais, Hereford, Simmental, Brown Swiss, and Holstein, as described by Nkrumah et al. [24]. All animals used in this study were managed according to the guidelines given by the Canadian Council on Animal Care [25], and the experimental procedures were approved by the Livestock Animal Care and Use Committee of the University of Alberta (no. AUP00000927).

All steers were offered an identical finishing diet, formulated with 75% barley grain, 20% barley silage, and 5% Killam (30%) Beef Supplement Pellets (Tag 849053; Hi-Pro Feeds, Westlock, AB, Canada) on an as-fed basis, ad libitum, and had free access to water. Feed intake was individually recorded during the 70-day experimental period, between April and August of each year, using an automated feeding system (GrowSafe Systems Ltd., Airdrie, AB, Canada), and the dry matter intake (DMI) was calculated based on moisture content of the total mixed ration. Initial body weight and average daily gain (ADG) for each steer were obtained from a linear regression of serial body weight (BW) measurements that were recorded over consecutive days at the beginning of the study and at approximately 14-day intervals during the feedlot test, and over two consecutive days at the end of feedlot experiment. The BW of AN, CH, and KC at the beginning of the feedlot experiment were 432.1 ± 8.6 kg, 534.4 ± 11.3 kg, and 407.2 ± 15.5 kg, respectively, for 2014, and 502.7 ± 15.3 kg, 529.2 ± 8.0 kg, and 416.6 ± 9.5 kg, respectively, for 2015. Metabolic body weight (MWT) was calculated as the midpoint BW^0.75^, where midpoint BW was computed as the sum of the initial BW of steers and the product of its ADG multiplied by half the number of days under the feedlot experiment. The individual RFI values were calculated based on DMI, ADG, and MWT, as described by Nkrumah et al. [26].

At the end of the feeding experiment, a total of 96 steers (48 heads each year) were selected based on their RFI ranking (high-RFI: value > 0.5; low-RFI: value < −0.5; see Table S1) and were slaughtered before feeding at Lacombe Research Centre (Agriculture and Agri-Food Canada, Lacombe, AB, Canada). Specifically, within each year, there were 16 steers belonging to each breed (*n* = 16 for AN, CH, and KC, respectively) and 8 steers belonging to each RFI category (*n* = 8 for Low-RFI and High-RFI, respectively) for each breed. For each steer, about 50 mL of rumen content sample (including rumen fluid and feed particles) were collected individually within 30 min of the animals being slaughtered. Samples were immediately snap-frozen using liquid nitrogen and then stored at −80 °C until further analyses.

### 2.2. Nucleic Acid Extractions and cDNA Synthesis

Total DNA was extracted from 0.3~0.5 g of the rumen content sample, collected from each animal using the repeated bead beating method, as described by Yu and Morrison [27]. The integrity of the DNA was verified by 1% agarose gel electrophoresis and the yield was measured using a NanoDrop Spectrophotometer ND−1000 (Thermo Fisher Scientific Inc., Wilmington, DE, USA). The total RNA was extracted from 0.3~0.5 g of the rumen content sample using Trizol reagent, following the procedures outlined by Li et al. [16]. The quantity of RNA was measured using a NanoDrop and the quality of the RNA was assessed using an Agilent 2100 Bioanalyzer (Agilent Technologies, Santa Clara, CA, USA). The cDNA of each sample was generated by reverse transcription via 1000 ng of total RNA, each using the iScript Reverse Transcription Supermix kit (Bio-Rad Laboratories Inc., Hercules, CA, USA).

### 2.3. Quantitative Real Time PCR Analysis

All DNA samples were diluted to 50 ng/µL as a template to quantify the total abundance of the 4 microbial groups, and 20 times diluted cDNAs were used as a template to quantify the total active population of the 4 microbial groups. The abundance and active population of the four targeted microbial groups was determined using quantitative real-time PCR (qRT-PCR), with four pairs of universal primers: total bacteria (U2-F: ACTCCTACGGGAGGCAG; U2-R: GACTACCAGGGTATCTAATCC) [28], total archaea (uniMet1-F: CCGGAGATGGAACCTGAGAC; uniMet1-R: CGGTCTTGCCCAGCTCTTATTC) [11], total protozoa (SSU-316 F: GCTTTCGWTGGTAGTGTATT; SSU-539 R: CTTGCCCTCYAATCGTWCT) [29], and total anaerobic fungi (Fungi-F: GAGGAAGTAAAAGTCGTAACAAGGTTTC; Fungi-R: CAAATTCACAAAGGGTAGGATGATT) [30], respectively. Standard curves were constructed using serial dilutions of purified plasmid containing the full-length 16 S rRNA gene of *Butyrivibrio hungatei* for total bacteria, the partial 16 S rRNA gene of *Methanobrevibacter sp*. strain AbM4 for total archaea, the partial 18 S rRNA gene of *Entodinium longinucleatum* for total protozoa, and the partial internal transcribed space (ITS) rRNA gene of *Punctularia strigosozonata* for total fungi. qRT-PCR was conducted using SYBR green chemistry (Fast SYBR Green Master Mix, Applied Biosystems, Carisbad, CA, USA) and the StepOnePlus Real-time PCR System (Applied Biosystems, Life Technologies Holdings Pte Ltd., Singapore) with a holding stage, a fast cycle, and then a melt curve section. The PCR programs were as follows: for bacteria, a holding stage of 95 °C for 5 min, followed by 40 cycles at 95 °C for 20 s and 60 °C for 30 s; for the rest of the three microbial groups, the starting temperature was 95 °C, but holding occurred for only 20 s, followed by 40 cycles at 95 °C for 3 s and 60 °C for 30 s. For melting curve detection, all were started from 95 °C for 15 s, followed by 60 °C for 1 min, and then the temperature was increased by 0.3 °C for every 20 s period, from 60 °C up to 95 °C, then kept at 95 °C for 15 s. The maker gene copies for each microbial group in the per-gram rumen content, at both DNA and RNA levels, were calculated using the equation described previously [11].

### 2.4. Statistical Analysis

The marker gene copies of four microbial groups at either the DNA or RNA level were respectively subjected to PCA analysis in R (version 4.1.2, R Core Team, Vienna, Austria), and the results were visualized by the ggbiplot package to illustrate the effects of breed and RFI. The marker gene copies were then log-transformed to further conduct parametric tests. To elucidate the dissimilarity of abundance and active population in the four microbial groups (in the rumen of steers fed with a finishing diet), the difference in the respective marker gene copies between two types of genetic resource (DNA/RNA) were compared using a paired t-test, and the difference (A) in the order of magnitude of the marker gene copies for each targeted microbial group, between DNA and RNA level, means that its abundance differs by 10 ^A^ times from its active population. The correlation between the four microbial groups at both the abundance and active population levels were also analyzed using the CORR program in SAS, with *p* < 0.05 declared as significant. To assess the effects of breed and RFI, as well as their interaction on the abundance (DNA level) and activity (RNA level) of each targeted microbial group, Log-transformed data were further analyzed using the mixed program in SAS (Version 9.4; SAS Institute, Inc., Cary, NC, USA), with the mixed effect model set as the following:

Y_ijk_ = μ + α_ik_ + β_jk_ + (α × β)_ijk_ + θ_k_ + ξ_ijk_

where μ is the intercept and ξ_ijk_ is the residual error term, and α_ik_, β_jk_, and (α × β)_ijk_ are the fixed effects of the *i*th beef breed (AN, CH, KC) and the *j*th RFI classification (high and low RFI) and their interaction, respectively. θ_k_ represents the effect of the sampling year. The sampling year was used as a random effect when differences between factor effects (breed, feed efficiency, and their interaction) were compared due to treatments being identical between the two years. The Student’s *t*-test was used for multiple comparisons, with the significance level defined as *p* < 0.05.

## 3. Results

### 3.1. Assessment of Four Targeted Microbial Groups in Rumen of Beef Steers

Regardless of the breed and RFI effects, the average abundance (at the DNA level) for the four microbial groups was 2.00 × 10^11^ (11.30 log) copies of bacterial 16 S rRNA gene per gram of rumen content, 3.63 × 10^9^ (9.56 log) copies of archaeal 16 S rRNA gene per gram of rumen content, 5.01 × 10^8^ (8.70 log) copies of protozoal 18 S rRNA gene per gram of rumen content, and 2.95 × 10^5^ (5.47 log) copies of fungal ITS gene per gram of rumen content. The average active population (at the RNA level) for the four microbial groups was 4.79 × 10^10^ (10.68 log) copies of bacterial 16 S rRNA gene per gram of rumen content, 1.55 × 10^9^ (9.19 log) copies of archaeal 16 S rRNA gene per gram of rumen content, 2.88 × 10^10^ (10.46 log) copies of protozoal 18 S rRNA gene per gram of rumen content, and 2.09 × 10^4^ (4.32 log) copies of fungal ITS gene per gram of rumen content. The abundance values for bacteria, archaea, and fungi were 4.17, 2.34, and 13.80 times higher (*p* < 0.01) than their active population values, respectively, while protozoal abundance was 58.88 times less (*p* < 0.01) than its active population (Table 1). Significant differences (*p* < 0.01) were observed between the average marker gene copies at the DNA and RNA levels for all four targeted microbial groups (Table 1).

Meanwhile, correlation analysis showed that there were significant positive correlations (*p* < 0.05) between the respective marker gene copy numbers of the four microbial groups at either the DNA or RNA level, except between bacteria and fungi at the DNA level (Table 2). Moderate positive correlations (r values ranged from 0.23 to 0.56) at the DNA level, and more robust correlations (r values ranged from 0.30 to 0.90) at the RNA level, were observed between the four microbial groups (Table 2). Specifically, high positive correlations between archaea and bacteria (r = 0.93) as well as between archaea and protozoa (r = 0.81, *p* < 0.01) at the RNA level (Table 2) were found.

### 3.2. Effects of Breed and RFI on the Abundance of the Four Microbial Groups in the Rumen

**Figure 1:**
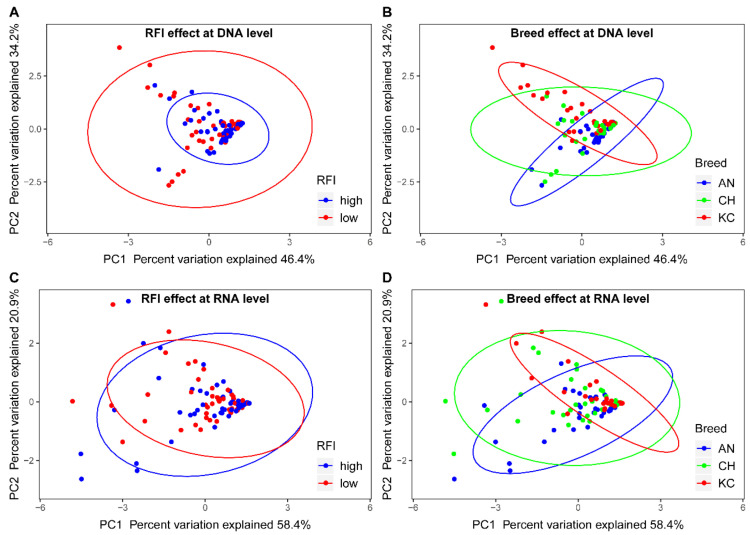
The similarity in rumen microbiota profiles for steers of either a different breed (AN: angus; CH: Charolais; KC: crossbred Kinsella composite) or with divergent RFI (high and low). PCA analysis was conducted on absolute copies of the targeted microbial groups at either the DNA (**A**,**B**) or RNA (**C**,**D**) level. The ovals in the plot are 95% inertia ellipses.

Differential abundance analysis, based on the log-transformed marker gene copies of targeted microbial groups at the DNA level, revealed that breed had a significant effect on the abundances of the targeted microbial groups (*p* < 0.01), except for archaea (*p* = 0.60) (Table 3). However, neither RFI nor the interaction of RFI and breed had an effect on the abundance of any microbial group (*p* > 0.05). Further PCA analysis revealed that principal components 1 and 2 together explained 80.6% of the total variation (Figure 1A,B), and the 95% inertia ellipse tended to separate according to breed (Figure 1B) but not to RFI (Figure 1A).

Specifically, as showed in Table 3, the protozoal abundance in the rumen of AN steers was 1.74 × 10^8^ (8.24 log) copies of 18 S rRNA gene per gram of rumen content, which was significantly lower (*p* < 0.05) than that of KC and CH steers, each with 1.20 × 10^9^ (9.08 log) and 7.24 × 10^8^ (8.86 log) copies of 18 S rRNA gene per gram of rumen content, respectively. In contrast, the bacterial abundance in the rumen of AN steers (2.57 × 10^11^ (11.41 log) copies of 16 S rRNA gene per gram of rumen content) was greater (*p* < 0.05) than that of the KC animals (1.26 × 10^11^ (11.10 log) copies of 16 S rRNA gene per gram of rumen content), but both of them had no difference (*p* > 0.05) compared with the rumen of the CH steers (1.74 × 10^11^ (11.24 log) copies of 16 S rRNA gene per gram of rumen content). Besides, KC steers (2.09 × 10^6^ (6.32 log) copies of fungal ITS gene per gram of rumen content) had a greater fungal abundance in the rumen than those in the rumen of AN steers (1.15 × 10^6^ (5.06 log) copies of fungal ITS gene per gram of rumen content) and CH (1.82 × 10^6^ (5.26 log) copies of fungal ITS gene per gram of rumen content) cattle (*p* < 0.05).

### 3.3. Effects of Breed and RFI on Active Populations of the Four Microbial Groups in the Rumen

Similarly, breed did have an effect on the active populations of the targeted microbial groups (*p* < 0.01), except for in fungi (*p* = 0.65) (Table 4), while no effect was observed for RFI and the interaction between RFI and breed (*p* > 0.05). The active populations of bacteria (5.75 × 10^9^ (9.76 log) copies of 16 S rRNA gene per gram of rumen content), archaea (2.29 × 10^8^ (8.36 log) copies of 16 S rRNA gene per gram of rumen content), and protozoa (1.05 × 10^10^ (10.02 log) copies of 18 S rRNA gene per gram of rumen content) in the rumen of the KC steers were significantly lower (*p* < 0.05) than those in the rumen of AN steers (1.32 × 10^11^ (11.12 log) copies of 16 S rRNA gene per gram of rumen content for bacteria, 3.80 × 10^9^ (9.58 log) copies of 16 S rRNA gene per gram of rumen content for archaea, and 7.41 × 10^10^ (10.38 log) copies of 18 S rRNA gene per gram of rumen content for protozoa, respectively) and those in the rumen of CH cattle (7.41 × 10^10^ (10.87 log) copies of 16 S rRNA gene per gram of rumen content for bacteria, 4.17 × 10^9^ (9.62 log) copies of 16 S rRNA gene per gram of rumen content for archaea, and 7.41 × 10^10^ (10.87 log) copies of 18 S rRNA gene per gram of rumen content for protozoa, respectively). In addition, the PCA plots demonstrated that principal components 1 and 2 together explained 79.3% of the total variation, and the 95% inertia ellipse also tended to separate according to breed (Figure 1D) yet almost overlapped between the high- and low-RFI groups (Figure 1C).

## 4. Discussion

As a rapid, reproducible, quantifiable nucleic acid-based molecular approach, qRT-PCR has become an alternative to the conventional culture-based method for determining microbial populations in different environments [31], which could potentially be considered as a trait that reflects rumen fermentation. Our results on the simultaneous assessment of the abundance (at the DNA level) and active populations (at the RNA level) of four microbial groups using qRT-PCR revealed significantly different outcomes for all groups when a different nucleic acid was used. It is known that when the numbers of marker genes are assessed at the DNA level, this may overestimate the bacterial populations, as the presence of DNA may have originated from dead or lysed cells [32]. Significant differences were found between the abundance and active populations in the rumen of these four microbial groups, suggesting that only DNA-based analysis may result in biased conclusions on rumen microbial populations.

Although small subunit RNA (e.g., 16 S, 18 S rRNA) genes are the most popular molecular markers for studying microbial diversity, composition, and abundance, it is important to be aware that numbers estimated based on marker gene copies can be biased due to different organisms containing varied copy numbers of small subunit RNA genes in their genomes [33]. As reported, copy numbers of ribosomal RNA genes were recorded at up to 15 [33,34,35] and 5 [36], ranging from 61 to 316,000 [36,37], and ranging from 60 to 220 [38] in bacterial, archaeal, protozoal, and fungal genomes, respectively. When marker gene copy numbers of targeted microbial groups at the DNA level were corrected using the ribosomal RNA gene copy numbers, as mentioned above, the estimated rumen microbial populations were 10^10^–10^11^ cells per gram of rumen content for bacteria, 10^9^ cells per gram of rumen content for archaea, 10^3^–10^7^ cells per gram of rumen content for protozoa, and 10^3^–10^4^ cells per gram of rumen content for fungi. These results are similar to previously reported populations, enumerated via both microscopic and culture-independence methods [29,39], suggesting that the PCR-based quantification of microbial populations is representative of the rumen microbiota of the steers.

A complex succession of microorganisms takes part in the cooperative catabolism of substrates in the rumen to perform the complete degradation of a feed substrate [18,40]. Knowledge of the relationships among the populations of different microbial groups may help to understand the interactions among them and their synergic roles that contribute to rumen fermentation. Correlations among bacteria, archaea, and protozoa were found at both the DNA and RNA level. In line with our results, Wallace et al. [41] also observed that there was a weak correlation between archaea and bacteria abundance levels in the rumen of beef steers. These further confirmed the interactions among bacteria, archaea, and protozoa. However, the identified higher correlation at the RNA level when compared to the DNA level in our study is the first suggestion that the interactions among rumen microbial groups mainly manifest at the activity level rather than the abundance level. For example, the active population of archaea was found to be highly correlated with those of bacteria and protozoa. In the rumen, archaea utilize carbon dioxide and hydrogen, which are the end products produced by many other microbes like protozoa, bacteria, and anaerobic fungi, to produce methane [42]. Methanogenesis is an important process that releases the partial pressure of hydrogen, which might inhibit the normal function of microbes as well as rumen fermentation [22]. Our results suggest that at the activity level, archaea are more associated with bacteria and protozoa and have a limited relationship with anaerobic fungi in the rumen of steers fed with a feedlot diet. To date, the function of fungi in a high-grain diet has not been intensely studied, which now warrants further investigation.

The current study also revealed that breed had a significant effect on rumen microbiota at both the abundance and active population level. Specifically, in the rumens of KC steers, a higher abundance of eukaryotes and a lower abundance of bacteria, as well as a higher abundance of fungi, were detected when compared to those in the rumen of AN and CH steers, respectively. Meanwhile, the active population of targeted ruminal microbial groups, except fungi in KC steers, was lower than that in AN and CH animals. These results confirmed that rumen bacteria and archaea in KC steers were distinct from those in AN and CH animals, not only at the compositional and functional levels but also at the abundance and active population levels [15]. Similar to our study, Paz et al. [43] suggested that Holstein and Jersey cows harbor different rumen bacterial communities based on 16 S rRNA gene sequencing analysis. Recently, Li et al. [15] also reported the effect of breed on active rumen bacteria and archaea. However, the effect of breed on rumen protozoa and fungi is not well known. Our results further highlight that the host’s breed has a significant influence on not only prokaryotes but also eukaryotes in the rumen. It has been speculated that host genetics could influence several biological functions in animals, for instance, eating frequency, feed intake, rumen size, and rumen passage rate [15]. In addition, the coevolution of microorganisms with their host might be one of the mechanisms explaining how the host’s breed can affect different rumen microbial groups [2]. However, compared with the AN steers, the significantly decreased active microbial populations, relative to the abundance in the rumen of the KC animals, might be attributed to a lower DMI (Table S1). It is notable that more abundance and less active populations of protozoa were observed in the rumen of the KC steers, combined with lower archaeal activity, suggesting that the composition of the protozoal community in KC steers might be different from the other two breeds. Future studies on the comparison of rumen microbial profiles at the compositional level are needed to verify our speculations.

Compared to bacteria, archaea, and fungi, the copy numbers of protozoa at the RNA level were markedly higher than at the DNA level, suggesting that the protozoa may have high activity in the rumens of beef steers fed with a high-grain diet. As an important part of the rumen ecosystem, rumen eukaryotic microbes contribute up to half of the rumen microbial biomass and also comprise hundreds of eukaryotic linkages. To date, more than 250 species of ciliates belonging to 1 class (Litostomatea), 2 orders (Vestibuliferida and Entodiniomorphida), 16 families, and at least 25 genera have been identified in the forestomach and large intestine of herbivorous animals [18,44]. Some Holotrich ciliates, like *Isotricha* and *Dasytricha,* play important roles in utilizing soluble sugars, whereas some Entodiniomorphid ciliates are capable of engulfing whole starch granules. As a result, the presence of protozoa controls the rate of carbohydrate fermentation, especially when large quantities of soluble carbohydrates are present in the diet [18]. Future studies on the taxonomic composition may help us to dissect the effect of breed on protozoa at the species level.

No significant differences in abundance or active population were found between high- and low-RFI cattle, which confirmed previous reports, where no difference was detected between the total bacteria [20] and total archaea populations [12]. Our team reports a difference between bacterial and archaeal composition [12,45], as well as between active bacterial and archaeal members [15] for high- and low-RFI KC cattle under the same feedlot diet. This, together with many other studies reporting a varied bacterial and archaeal composition in animals with different feed efficiencies [9,13,46,47], suggests that bacterial and archaeal composition, rather than population, play a decisive role in contributing to the RFI, which may also be the case for rumen protozoa and fungi.

## 5. Conclusions

This study assessed the total abundance and active populations of four ruminal microbial groups in the rumen of beef steers of divergent RFIs and breeds. There was a significant difference in abundance and active population levels in all of the targeted microbial groups, and the breed had a significant effect on all microbial groups. The results reveal that breed (host genetics) affects rumen eukaryotes, suggesting that the role of host genetics together with diet can regulate the rumen microbiota as a whole. These findings indicate that some rumen microbial communities could be influenced by the host breed, highlighting a potential to manipulate desirable and efficient rumen microbiota (especially eukaryotes) using a breeding strategy. In addition, the significant difference in microbial populations quantified by DNA- and RNA-based methods implied that more caution should be considered when interpreting the outcomes obtained from culture-independent molecular-based methods. Furthermore, the active population of protozoa was remarkably higher relative to its abundance, implying that it might play a pivotal role in metabolic activity under a feedlot high-grain diet. Although rumen microbial abundance and active populations presented no statistical difference between the steers with a divergent RFI in this study, future work employing high-throughput next generation sequencing is needed to further elucidate the biodiversity of rumen eukaryotes, especially protozoa, and their potential role in beef cattle feed efficiency.

## Figures and Tables

**Table 1:** Log-transformed marker gene copies (means ± SD, g^−^^1^ rumen content) of the targeted microbial groups at the DNA and RNA level.

Item	DNA	RNA	RNA-DNA	Fold Change (RNA/DNA)	*p* Value
Bacteria	11.30 ± 0.36 ^a^	10.68 ± 0.36 ^b^	−0.62 ± 0.11	1/4.17	<0.01
Archaea	9.56 ± 0.24 ^a^	9.19 ± 0.24 ^b^	−0.37 ± 0.10	1/2.34	<0.01
Protozoa	8.70 ± 0.27 ^b^	10.46 ± 0.27 ^a^	1.77 ± 0.15	58.88	<0.01
Fungi	5.47 ± 0.51 ^a^	4.32 ± 0.51 ^b^	−1.14 ± 0.10	1/13.80	<0.01

^a, b^ Means with different superscripts in each row were significantly different.

**Table 2:** Person correlation matrix among the four microbial groups at the population (DNA) and activity (RNA) level.

Item	Mean	SD.	Bacteria	Archaea	Protozoa	Fungi
DNA						
Bacteria	11.30	0.41	1			
Archaea	9.56	0.37	0.57 ^a^	1		
Protozoa	8.70	1.03	0.44 ^a^	0.23 ^b^	1	
Fungi	5.47	1.40	0.17 ^c^	0.26 ^b^	0.54 ^a^	1
RNA						
Bacteria	10.68	1.11	1			
Archaea	9.19	1.04	0.93 ^a^	1		
Protozoa	10.46	1.40	0.68 ^a^	0.81 ^a^	1	
Fungi	4.32	1.06	0.35 ^a^	0.30 ^a^	0.43 ^a^	1

Note: ^a^ *p* < 0.01; ^b^ *p* < 0.05; ^c^ *p* > 0.05.

**Table 3:** Effects of breed and divergent RFIs on the log-transformed marker gene copies (g^−1^ rumen content) of the targeted microbial groups at the DNA level.

Items	Breed			RFI		*p* Value		
	Angus	Charolais	Kinsella	High	Low	Breed	RFI	Breed × RFI
Bacteria	11.41 ± 0.12 ^a^	11.24 ± 0.18 ^ab^	11.10 ± 0.08 ^b^	11.22 ± 0.12	11.28 ± 0.11	<0.01	0.53	0.23
Archaea	9.55 ± 0.12	9.52 ± 0.18	9.44 ± 0.07	9.46 ± 0.12	9.55 ± 0.11	0.60	0.20	0.47
Protozoa	8.24 ± 0.30 ^b^	8.86 ± 0.45 ^a^	9.08 ± 0.19 ^a^	8.62 ± 0.30	8.82 ± 0.29	<0.01	0.27	0.53
Fungi	5.06 ± 0.31 ^b^	5.26 ± 0.42 ^b^	6.32 ± 0.24 ^a^	5.36 ± 0.29	5.75 ± 0.28	<0.01	0.13	0.80

^a, b^ Means with different superscripts in each row were significantly different (*p* < 0.05); RFI, Residual Feed Intake.

**Table 4:** Effects of breed and divergent RFI on the log-transformed marker gene transcript copies (g^−^^1^ rumen content) of the targeted rumen microbial groups at the RNA level.

Item	Breeds			RFI		*p* Value		
	Angus	Charolais	Kinsella	High	Low	Breeds	RFI	Breeds × RFI
Bacteria	11.12 ± 0.27 ^a^	10.87 ± 0.40 ^a^	9.76 ± 0.19 ^b^	10.60 ± 0.26	10.57 ± 0.26	<0.01	0.86	0.92
Archaea	9.58 ± 0.16 ^a^	9.62 ± 0.16 ^a^	8.36 ± 0.16 ^b^	9.20 ± 0.13	9.18 ± 0.13	<0.01	0.91	0.95
Protozoa	10.38 ± 0.29 ^a^	10.87 ± 0.36 ^a^	10.02 ± 0.25 ^b^	10.39 ± 0.26	10.47 ± 0.26	<0.10	0.78	0.77
Fungi	4.35 ± 0.35	4.38 ± 0.54	4.62 ± 0.21	4.40 ± 0.35	4.50 ± 0.34	0.65	0.61	0.95

Note: Means with different superscripts in each row were significantly different (*p* < 0.05); RFI, Residual Feed Intake.

## Data Availability

Data is contained within the article or supplementary material.

## Supplementary Materials

The following supporting information can be downloaded at: https://www.mdpi.com/article/10.3390/ani12151966/s1, Table S1: The production performance of beef cattle between High- and Low-RFI groups within each breed.
