# Supplementary material for: The Effects of Breed and Residual Feed Intake Divergence on the Abundance and Active Population of Rumen Microbiota in Beef Cattle
Source: Animals (Basel). 2022 Aug 3;12(15):1966. doi: 10.3390/ani12151966 (PMC9367312; doi:10.3390/ani12151966)
Supplement: Supplementary file 1 [file animals-12-01966-s001.zip › animals-1826240-supplementary.pdf]

Table S1. Production performance of various breeds of beef steers with divergent RFI

Item		Angus	Charolais	Kinsella Composite
RFI kg/d	High-RFI	1.10±0.10	0.96±0.08	1.04±0.14
	Low-RFI	-0.66±0.10	-0.89±0.08	-0.98±0.14
	P value	**	**	**
DMI kg/d	High-RFI	12.09±0.73a	11.63±0.45a	11.07±0.81a
	Low-RFI	10.87±0.73b	9.89±0.45b	9.33±0.80b
	P value	**	**	**
ADG kg/d	High-RFI	1.59±0.07	1.60±0.06	1.46±0.06
	Low-RFI	1.85±0.07	1.67±0.06	1.61±0.06
	P value	**	n.s.	*
FCR	High-RFI	7.88±0.51	7.34±0.19	7.57±0.35
	Low-RFI	5.90±0.51	5.99±0.19	5.89±0.34
	P value	**	**	**

Notes: n.s. not significant; * $P<0.05$; ** $P<0.01$; RFI: Residual feed intake; DMI: Dry matter intake; ADG: Average daily gain; FCR: Feed conversion ratio.
